# Perfusion imaging combined with coronary calcium scoring. A step further towards individualized medicine?

**DOI:** 10.1016/j.ijcha.2021.100845

**Published:** 2021-07-28

**Authors:** Lars Frost, Thomas Gerds, Nicklas Vinter

**Affiliations:** Diagnostic Centre, Silkeborg Regional Hospital, Silkeborg, Denmark; Department of Clinical Medicine, Aarhus University, Aarhus, Denmark; Section of Biostatistics, University of Copenhagen, Copenhagen, Denmark; Diagnostic Centre, Silkeborg Regional Hospital, Silkeborg, Denmark; Department of Clinical Medicine, Aarhus University, Aarhus, Denmark; Danish Center for Clinical Health Services Research, Aalborg University, Aalborg, Denmark

Coronary calcification is an important marker of atherosclerotic coronary artery disease. Traditionally, the use of non-contrast cardiac computed tomography and the Agatston score method has quantified a coronary artery calcium score (CACS) that reflects the level of calcification [1]. Several studies have demonstrated that the CAC score is associated with increased hazard rates of major cardiovascular events, and CAC has an important role in risk stratification [2]. Advancements in imaging technologies have enabled examination of newer and perhaps better ways of quantifying the level of coronary calcification. Combining anatomical and functional data in the evaluation of patients could have the potential to improve test quality.

In this issue of *IJC Heart & Vasculature*, Trpkov et al. examined “the independent prognostic value” of a visually estimated CAC score (VECACS) in patients with suspected or known coronary artery disease [3]. VECACS was quantified in 4720 patients in conjunction with single photon emission computed tomography myocardial perfusion imaging (SPECT-MPI), and the patients were followed for major adverse cardiac events (MACE). MACE included all-cause mortality, acute coronary syndrome, or revascularization >90 days after SPECT-MPI. Multivariable Cox regression adjusted for age, male sex, diabetes, dyslipidemia, smoking status, history of CAD, chronic heart failure, stroke, chronic kidney disease, exercise stress, inpatient status, summed stress score, summed rest score, summed difference score, and left ventricular ejection fraction showed an increased MACE hazard rate associated with VECACS categories compared to absent (equivocal: adjusted hazard ratio [HR] 2.54, 95% CI 1.45–4.45, p = 0.001, present (adjusted HR 2.44, 95% CI 1.74–3.42, p < 0.001, extensive adjusted HR 3.47, 95% CI 2.41–5.00, p < 0.001). Addition of VECACS to the multivariable Cox regression model was evaluated with improper statistical tools.

Before recommending a new imaging technique in the risk stratification of patients with known or suspected coronary artery disease, we must consider methodological applications and relevance. A distinction between etiological research and medical prediction is important as the approach and clinical consequences of the results are different [4]. Briefly, etiological research aims to explain a biological system, in order to identify the modifiable risk factors and interventions that may lead to prevention of disease. In contrast, prediction models provide personalized predictions of the absolute risk of MACE within a prespecified time period, e.g., 5 years (or within 10 years), which can be used to risk-stratify patients and to guide therapeutic decision-making.

A limitation of the study by Trpkov et al. [3] is the lack of a clear distinction between the etiological perspective and the aim to predict the personalized risks of MACE. It is hard to imagine that CAC *per se* can cause plaque rupture leading to acute myocardial infarction or that CAC *per se* can cause coronary luminal obstruction leading to angina pectoris. CAC is most likely a biomarker that reflects coronary plaque burden in one single measure that can be useful in the prediction of MACE [5].

An important limitation of the study by Trpkov et al. [3] is the lack of statistical rigor. It is well known that a significant hazard ratio does not translate into added predictive power and hence it is not possible to conclude that a variable is an independent predictor based on the significance test of the hazard ratio in a multivariable Cox regression [6], [7]. Additionally, the time point at which the prediction is given to the patient and the prediction horizon needs to be well defined in order to discuss a medical risk prediction model [8]; there is an important difference between the risk of MACE within 1 year and within 5 years from now, especially in the elderly patients. The absolute risk is essential to risk-stratify patients and to guide shared decision-making for reducing the risk of MACE. Patients with a high absolute risk of MACE for instance at 1 year could be subject to extensive follow-up visits with focus on reducing modifiable risk factors and optimizing or intensifying medical therapy.

The authors examined the predictive performance using the Net Reclassification Index (NRI). The NRI has been suggested to measure the increasing prognostic value when adding a new predictor to an existing statistical prediction model [9]. However, NRI is not a proper measure of performance because adding a marker that is not associated at all with the outcome can lead to increased NRI [10]. The Brier score is a proper measure of prediction performance which assesses discrimination and calibration at the same time [8]. However, without data splitting (cross-validation) it is not possible to interpret the difference of the added value of VECACS in terms of the Brier score because the model which includes VECACS spends 3 more parameters for the 4 VECACS categories than the model without VECACS. Hence, the apparent improvement could simply be explained by overfitting of the data.

Despite these concerns about the statistical methods, CAC is an important biomarker whose place in individualized medicine may expand. Adding CAC to the Multi-Ethnic Study of Atherosclerosis (MESA) risk score improved discrimination, which was also found when the model was externally validated [11]. Recently, the 2018 AHA/ACC Guideline on the Management of Blood Cholesterol has added the use of CAC for guiding initiation of statin therpy as primary prevention [12].

Before we can implement routine evaluation of VECACS for patients with suspected or known CAD, more evidence is needed. First, the prediction model needs a clearly defined clinical framework in which it should be applied. Next, one should study internal validation using repeated data splitting and external validation using data collected in a comparable framework elsewhere or later in calendar time; eventually one could study the clinical utility of the prediction mode by means of randomized clinical trials [13]. Such trials could provide valuable evidence to omit perfusions scans in chest pain patients with a CAC of 0. We need evidence to abstain from lipid-lowering treatment and antiplatelet treatment in CAC of 0. We also need evidence for use of PCSK9 inhibitors for primary and secondary prevention among subjects with a high CAC value. Even though correlation has been found, no studies have thus far compared the predictive performance of CAC as measured by Trpkov et al. [3] versus the Agatston method, although the Agatston score is usually categorized in prognostic as well as in predictive studies. Nonetheless, Trpkov and colleagues [3] should be commended for using the unique imaging data to inspire and motivate further studies on risk prediction and newer methods for quantifying coronary calcification.

## Conflict of interest statement

The authors report no relationships that could be construed as a conflict of interest.
